# From wrist to research: Harnessing smartwatch data for equitable cardiovascular research

**DOI:** 10.1371/journal.pmed.1005260

**Published:** 2026-09-28

**Authors:** Manpreet Kaur, on behalf of the PLOS Medicine Staff Editors

**Affiliations:** Public Library of Science, Cambridge, United Kingdom

## Abstract

Cardiovascular disease is a leading cause of death, and progress in reducing mortality has stalled, calling for more research. In this Editorial, Manpreet Kaur discusses the opportunity for harnessing cardiovascular data from smartwatches, and the ethical and logistical barriers to ensuring smartwatch-derived data informs equitable and actionable cardiovascular research.

## Teaser

Cardiovascular disease is a leading cause of death, and progress in reducing mortality has stalled, calling for more research. An opportunity lies in harnessing cardiovascular data from smartwatches. However, ethical and logistical barriers need to be overcome to ensure smartwatch-derived data is utilised to inform equitable and actionable cardiovascular research.

Cardiovascular disease (CVD) is a leading cause of mortality. Worldwide estimates show 17 million deaths were CVD-related in 2019 and this is expected to rise to 23.3 million by 2030 [1]. Decades of progress had reduced cardiovascular mortality rates, but recent evidence suggests these improvements are stagnating, with age-standardised CVD mortality increasing in some populations, predominantly in low- and middle-income countries (LMICs) [2]. These findings highlight the need for continued CVD research. Increasingly, large-scale databases containing cardiovascular data from hundreds of thousands of participants, such as UK Biobank, the All of Us Research Program, and the China Kadoorie Biobank, are being used to inform research in areas such as the development of predictive models and drug repurposing [1]. However, these typically rely on single-country data and participant pools that are not representative of the general population, limiting generalisability and research effectiveness [3].

A new method of longitudinal cardiovascular data collection could overcome the geographical barrier of biobanks and potentially allow for the establishment of a global cardiovascular database: smartwatches and digital wearables. Nearly a quarter of the world’s population owns a smartwatch and while primarily used for personal tracking, the clinical potential of smartwatch data is being realised. Every day, these devices track various heart-related metrics including heart rate (HR), HR variation, blood pressure and sleep quality. Studies have shown that collection of metrics such as these can lead to diagnosis of conditions such as cardiomyopathy [4] and atrial fibrillation [5], among others, giving patients the opportunity for early intervention. Meanwhile, an observational clinical study reported high concordance between smartwatch-derived HR variation data compared with high-resolution electrocardiogram-derived data in patients with CVD, demonstrating its accuracy and diagnostic potential [6]. As wearables become increasingly accurate and diversified in their measurements, research facilities and smartwatch companies have started partnering to collate these data and maximise their research potential. The British Heart Foundation (BHF) Data Science Centre explored how to integrate smartphone and wearable information into a resource that can link to participants’ National Health Service records and to cardiovascular outcomes [7]. Apple has collaborated with the American Heart Association and Brigham and Women’s Hospital in the Apple Heart & Movement Study, which collects individual-level data and explores the relationship between activity, wellness, and health [8]. Meanwhile, Singapore has launched Health Insights Singapore (hiSG), in which participants are provided with a smartwatch to partake in a study assessing the health behaviours and lifestyles of residents [9]. While such initiatives show promise, there are still barriers to overcome to maximise their potential utility, including ensuring equitable participation and representation, as well as appropriate standardisation, integration and storage of individuals’ cardiovascular data.

While research using patient data is conducted under strict ethical guidelines, data collected by smartwatch companies is likely geared toward commercial and profit-oriented goals. If the data were to be used for research purposes, companies may be required to adhere to more stringent ethical guidelines—particularly pertaining to confidentiality—which could be challenging to implement if commercial and research interests conflict. Such collaborations are possible, however, as demonstrated by the Apple Heart & Movement Study, which incorporates participant health records by allowing users to share their records in a smartphone app; although only 10% of users were able to do so due to difficulties in interoperability between health records and smartwatch apps, highlighting ongoing issues around data standardisation and sharing [10]. This is further complicated when considering the integration and standardisation of data across multiple smartwatch manufacturers to maximise the number of participants.

Generally, modern smartwatches use photoplethysmography (PPG), a low-cost optical technique that detects blood volume changes in the skin’s microvasculature to obtain direct HR measurements, but apply different, largely proprietary additional sensors and algorithms to obtain derived metrics such as resting HR, requiring standardisation between different devices. Research also shows that PPG may not work as effectively on darker skin tones due to melatonin production, calling for correction algorithms and additional standardisations to be applied to ensure the data remains accurate and representative [11]. Overcoming these barriers will require collaborations between smartwatch manufacturers, researchers and health databases, although it is important to note that increasing the number of stakeholders that have access to patient health records also increases privacy concerns that will need to be managed.

A key challenge for database creation is the representativeness of populations and equitable access to the resultant research. A scoping review found that wearables can help reduce CVD burden in LMICs, but that adoption of them was hindered by factors such as technological literacy, cost and cultural considerations [12]. As smartwatches become more sophisticated, and potentially less user-friendly, ownership in older individuals (a population at high risk of CVD) may be deterred [12,13]. More sophisticated smartwatches also come with a bigger price tag, making affordability another divisive barrier and causing disparities in the socioeconomic backgrounds represented in databases. In 2019, 31% of US households that purchased a smartwatch had an income of>$75,000 whilst 12% of households earning <$30,000 owned one [14]. Individuals with lower incomes might also have less expensive or older models that might not track heart-related biometrics as comprehensively or as accurately as newer models. Representation of different ethnicities is also an issue. In a public survey conducted by the BHF to gauge which smartwatch data would have the most utility for CVD research, of the 194 respondents, only 6% identified as non-white [7]. While this doesn’t reflect the demographic breakdown of smartwatch owners, it points towards the risk of research being informed by and tailored towards particular demographics, entrenching health inequities. To prevent this, researchers and smartwatch manufacturers could provide subsidised smartwatches, as is being done in the hiSG initiative, with particular efforts for subsidies and educational campaigns in LMICs, and training for individuals who are willing to participate but face limitations.

Ownership and access to smartwatch data is dictated by the terms and conditions (T&Cs) that individuals agree to either upon purchasing a wearable device or using associated apps. While the most popular smartwatch manufacturers maintain that consumers own their data and can delete it at any time, the terms also usually specify that the company can control and share the data. In the Apple Heart & Movement Study, data access is granted to Apple, Brigham and Women’s Hospital, the American Heart Association, and the Research Studies Support Center. However, access to international databases would need to comply with research policies from multiple countries and consumer disclosure laws on how the data would be used by both manufacturers and research stakeholders. Consumers will likely need reassurance of its safety, particularly pertaining to involuntary surveillance and medicalisation, issues that will need to be communicated to consumers by concise and accessible means and not, for example, buried in overly lengthy T&Cs.

Mapping longitudinal cardiovascular data from smartwatches could provide insights into the early physiological changes that precede the onset of CVD, potentially improving disease prediction and opportunities for early intervention. Connecting biometric data to cardiovascular outcomes, and integrating it into researcher-accessible databases may accelerate insights into CVDs, such as arrhythmia, hypertension, heart failure, and ischaemic events [10], leading to treatment optimisation [7]. The hope is that these research efforts, by improving prediction, diagnosis and early intervention, will reverse the stagnated progress in decreasing global CVD mortality rates [2]. However, it is imperative that smartwatch manufacturers and researchers work collaboratively to build globally representative databases, maximise their utility, and prioritise equitable research goals.

## Abbreviations

BHF: British Heart Foundation
CVD: Cardiovascular disease
hiSG: Health Insights Singapore
HR: heart rate
LMICs: low- and middle-income countries
